# Редкий случай АКТГ-продуцирующей медуллярной карциномы щитовидной железы

**DOI:** 10.14341/probl13512

**Published:** 2025-07-22

**Authors:** Н. И. Тимофеева, Р. А. Черников, И. В. Слепцов, В. Ф. Русаков, Д. В. Реброва, С. Л. Воробьев, Т. С. Придвижкина, А. А. Семенов, М. А. Алексеев, А. Ю. Куликов

**Affiliations:** Клиника высоких медицинских технологий им. Н.И. Пирогова; Клиника высоких медицинских технологий им. Н.И. Пирогова; Клиника высоких медицинских технологий им. Н.И. Пирогова; Клиника высоких медицинских технологий им. Н.И. Пирогова; Клиника высоких медицинских технологий им. Н.И. Пирогова; Национальный центр клинической морфологической диагностики; Клиника высоких медицинских технологий им. Н.И. Пирогова; Клиника высоких медицинских технологий им. Н.И. Пирогова; Клиника высоких медицинских технологий им. Н.И. Пирогова; Клиника высоких медицинских технологий им. Н.И. Пирогова

**Keywords:** медуллярная карцинома щитовидной железы, эндогенный гиперкортицизм, АКТГ-эктопированный синдром, АКТГ-продуцирующая нейроэндокринная опухоль

## Abstract

Медуллярная карцинома является редким агрессивным видом рака щитовидной железы. Медуллярная карцинома щитовидной железы относится к нейроэндокринным опухолям, в связи с чем может обладать возможностью косекреции различных пептидных субстанций и гормонов. В работе описан уникальный клинический случай пациента с развитием тяжелого эндогенного гиперкортицизма вследствие гиперпродукции адренокортикотропного гормона (АКТГ) медуллярной карциномой щитовидной железы. Пациент мужчина, 39 лет, с уровнем кальцитонина более 4000 пг/мл (<11,8), с узлом в щитовидной железе и множественными метастазами в центральные и боковые шейные лимфоузлы. Уровень калия составлял 1,34 ммоль/л (3,5–5,1). Отмечалась гиперкортизолемия до 1613,2 нмоль/л (185–624) при повышенном уровне АКТГ до 24,7 пмоль/л (1,03–10,74). После коррекции водно-электролитных нарушений ему выполнена операция — тиреоидэктомия, центральная и боковая шейная лимфаденэктомия. В послеоперационном периоде кальцитонин снизился до 126 пг/мл (<11,8), уровни кальция и паратгормона оставались в норме. Уровень калия нормализовался без дополнительной фармакологической поддержки. В послеоперационном периоде развилась клиническая картина острой надпочечниковой недостаточности с резким снижением уровней кортизола и АКТГ крови, что потребовало назначения заместительной гормональной терапии большими дозами глюкокортикоидов. При морфологическом исследовании подтверждена медуллярная карцинома с множественными метастазами в шейные лимфоузлы, а также с продукцией АКТГ опухолевыми клетками. Таким образом, представлен редкий случай успешного лечения пациента с АКТГ-продуцирующей медуллярной карциномой щитовидной железы.

## Abstract (en)

Medullary thyroid carcinoma is a rare aggressive tumor of thyroid gland. Due to its neuroendocrine origine medullary thyroid carcinoma can be the source of ectopic production of different peptides and hormones. We describe a unique case of severe ACTH-dependent Cushing’s syndrome due to ectopic corticotropin production by medullary thyroid carcinoma in a male patient of 39 years old with calcitonin level more than 4000 pg/ml (<11,8), thyroid nodule, multiple neck jugular and central lymphnodes. The potassium level was 1,34 mmol/l (3,5–5,1), hypercortisolemia up to 1613,2 nmol/l (185–624) with elevated level of ACTH up to 24,7 pmol/l (1,03–10,74). After the correction of water and electrolytes disorders an operation was performed — thyroidectomy, central and lateral neck dissection. Postoperative calcitonin decreased to 126 pg/ml (<11,8), calcium and parathormone blood levels remained normal. The potassium level didn’t decrease without any pharmacological treatment. There was a sharp fall in postoperative blood levels of cortisol and ACTH. The hormonal replacement treatment with hydrocortisone was induced. Morphological examination showed medullary carcinoma with ACTH production in tumor nodule, with metastatic neck lymph nodes. Thus this is a rare case and successful treatment of patient with severe hypercortisolism by thyroidectomy and neck lymphnodes dissection.

## СПИСОК СОКРАЩЕНИЙ

## АКТУАЛЬНОСТЬ

Нейроэндокринные опухоли (НЭО) — гетерогенная группа новообразований, происходящих из нейроэндокринных клеток эмбриональной кишки, обладающих биологически активными свойствами [1].

Медуллярная карцинома щитовидной железы (МКЩЖ) относится к редким нейроэндокринным опухолям парафолликулярных С-клеток щитовидной железы (ЩЖ). Основными опухолевыми маркерами МКЩЖ являются кальцитонин и раковый эмбриональный антиген (РЭА) [2]. В связи с нейроэндокринным происхождением С-клетки ЩЖ также могут секретировать некоторые другие биоактивные пептиды, что в редких случаях приводит к развитию паранеопластических синдромов.

Эндогенный гиперкортицизм — симптомокомплекс клинических проявлений, отражающий избыточную секрецию кортизола опухолью надпочечника (синдром Иценко-Кушинга) или за счет вторичной стимуляции надпочечника адренокортикотропным гормоном (АКТГ) вследствие опухоли гипофиза (болезнь Иценко-Кушинга) или АКТГ-секретирующей опухоли другой локализации [3]. АКТГ-эктопированный синдром — это симптомокомплекс гиперкортицизма, развивающийся вследствие избыточной продукции АКТГ опухолью внегипофизарной локализации [3]. АКТГ-эктопированный синдром отличается более злокачественным течением и худшим прогнозом в сравнении с болезнью Иценко-Кушинга. Наиболее частая локализация (66%) НЭО — желудочно-кишечный тракт; около 30% НЭО встречаются в бронхопульмональной системе. Еще реже НЭО с гиперпродукцией АКТГ могут быть обусловлены другими опухолями: тимуса, надпочечников, поджелудочной железы, яичников, предстательной железы и почек [4].

Развитие гиперсекреции АКТГ-клетками МКЩЖ ассоциировано с агрессивным течением и худшим прогнозом [5]. Всего, по данным мировой литературы, за последние 65 лет описано около 100 случаев пациентов с диагнозом «АКТГ-эктопированный синдром с эктопической продукцией АКТГ-медуллярной карциномой щитовидной железы» [6][7][8], из них в России — 1 случай [5].

## КЛИНИЧЕСКИЙ СЛУЧАЙ

Пациент Г., 39 лет, обратился для плановой госпитализации в отделение эндокринной хирургии Клиники высоких медицинских технологий им. Н.И. Пирогова Санкт-Петербургского государственного университета (КВМТ СПбГУ) с диагнозом «Медуллярная карцинома щитовидной железы» для планового оперативного лечения. В связи с тяжестью состояния находился в инвалидной коляске в силу тетрапареза, доставлен отцом. При поступлении предъявлял жалобы на выраженную общую и мышечную слабость, онемение конечностей, интенсивный болевой синдром без четкой локализации (таз, спина, шея), светобоязнь. Сбор анамнеза затруднен из-за тяжести состояния и интеллектуально-мнестических нарушений, однако удалось выяснить следующее: около пяти лет назад заметил припухлость в области шеи. За медицинской помощью по этому поводу не обращался. За три года до поступления в клинику неоднократно находился на стационарном лечении по поводу перенесенной закрытой черепно-мозговой травмы, дренирования хронической субдуральной гематомы. За 5 месяцев до поступления в клинику выявлена полисегментарная пневмония на фоне новой коронавирусной инфекции с последующим развитием вторичного гидроторакса. За последние 3 месяца отмечалось быстрое прогрессирование неврологической симптоматики вплоть до развития тетрапареза. Примерно в этот период зафиксирована гипокалиемия до 2,7 ммоль/л (3,5–5,1), однако дальнейшее обследование для установления причин гипокалиемии и лечение с целью коррекции электролитных нарушений не проводились. Выявлен высокий уровень кальцитонина — 4001 пг/мл (<11,8) и РЭА — 70 нг/мл (<5). При ультразвуковом исследовании (УЗИ) визуализирован узел ЩЖ размером до 5 см. При цитологическом исследовании после тонкоигольной аспирационной биопсии (ТАБ) установлено наличие МКЩЖ. Кроме того, в медицинской документации указано на наличие сахарного диабета 2 типа (СД2), инсулинопотребного, с неизвестной давностью начала и схем сахароснижающей терапии.

## Результаты физикального, лабораторных и инструментальных исследований

При объективном осмотре пациента в приемном отделении: рост — 168 см, вес — 56 кг (ИМТ — 20,3 кг/м²). Температура — 35,6. Состояние тяжелое, обусловлено нарушением сознания на уровне сильного оглушения, синдромом системной воспалительной реакции с возможным сепсисом, интоксикацией, паранеопластическим синдромом. Реакция зрачков на свет сохранена. Отмечаются положительные менингеальные симптомы. Сухожильные рефлексы угнетены (тетрапарез). Кожные покровы землистой окраски, сухие. Тургор снижен. Кожа пергаментная, вены не контурируются. Лицо гиперемировано. На бедрах и в нижних отделах живота бледно-фиолетовые стрии. На нижних конечностях отеки, трофические язвы; на крестце — пролежни (рис. 1). Частота сердечного ритма — 84 уд./мин., пульс ритмичный, нитевидный. Артериальное давление 70 и 30 мм рт.ст. Частота дыхательных движений — 22–25 в 1 минуту. Дыхание жесткое, хрипов нет. Перистальтика ослабленная. Информации о стуле и диурезе нет.

**Figure:**
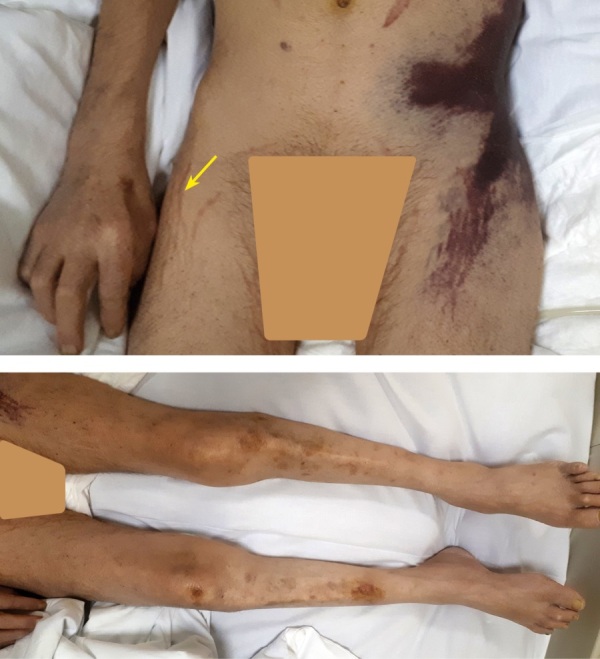
Рисунок 1. Множественные обширные кровоподтеки, подкожные гематомы и стрии.

По данным срочного анализа крови в приемном отделении: калий — 0,92 ммоль/л (3,5–5,1), ионизированный кальций — 0,84 ммоль/л (1,12–1,32), натрий — 139 ммоль/л (135–145), хлориды — 96,8 ммоль/л (98–107), кальцитонин — более 2000 пг/мл (<11,8), РЭА — 139,51 нг/мл (<5), прокальцитонин — 73,88 нг/мл (<0,07). Пациент госпитализирован в экстренном порядке в отделение реанимации для проведения интенсивной терапии.

При компьютерной томографии (КТ) шеи с внутривенным контрастированием: правая доля ЩЖ увеличена в размерах за счет образования до 5 см, нижний полюс опускается до грудинного конца ключицы. Многочисленные увеличенные боковые шейные и паратрахеальные лимфоузлы размером до 2,5 см с патологическим накоплением контраста (рис. 2).

**Figure:**
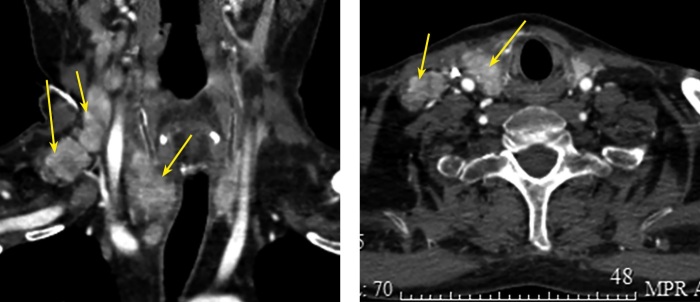
Рисунок 2. КТ шеи с в/в контрастом: образование правой доли ЩЖ — до 5 см, множественные центральные и боковые шейные лимфатические узлы с патологическим накоплением контраста.

При КТ грудной клетки свежих инфильтративных изменений не выявлено. В S3 левого легкого — уплотнение легочной ткани вытянутой формы — 2,5х1,8 см, накапливающее контрастное вещество (рис. 3). Лимфоузлы легких и корней легких не увеличены, структурно не изменены.

**Figure:**
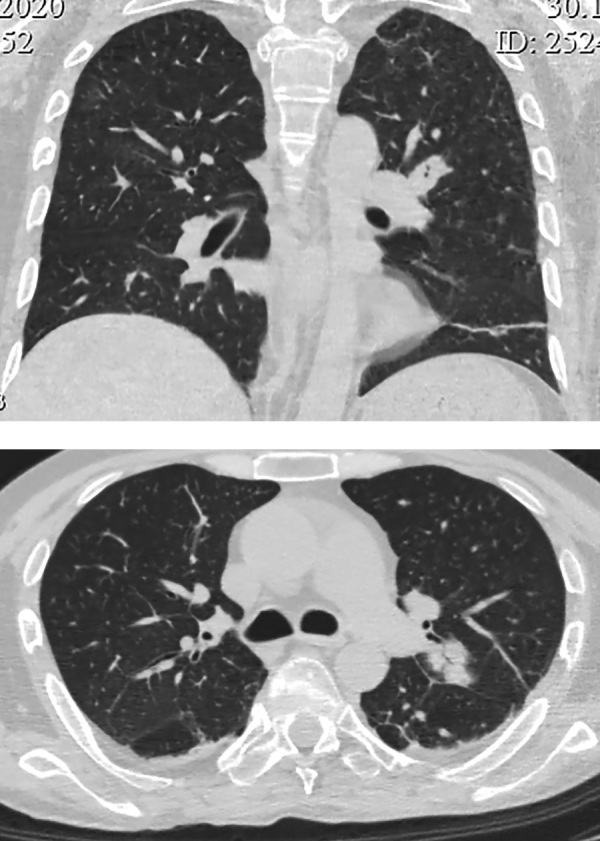
Рисунок 3. КТ органов грудной клетки: в S3 левого легкого уплотнение легочной ткани вытянутой формы — 2,5х1,8 см, накапливающее контрастное вещество.

Локальное уплотнение легочной ткани было расценено либо как метастаз опухоли ЩЖ, либо как первичная опухоль легкого. При КТ брюшной полости новообразований не выявлено. Визуализированы признаки пареза кишечника (рис. 4), диффузная гиперплазия обоих надпочечников — толщина правого увеличена до 1,4 см, левого — до 1,6 см (рис. 5). При КТ головного мозга выявлены признаки выраженной атрофии коркового вещества. Область турецкого седла не изменена.

**Figure:**
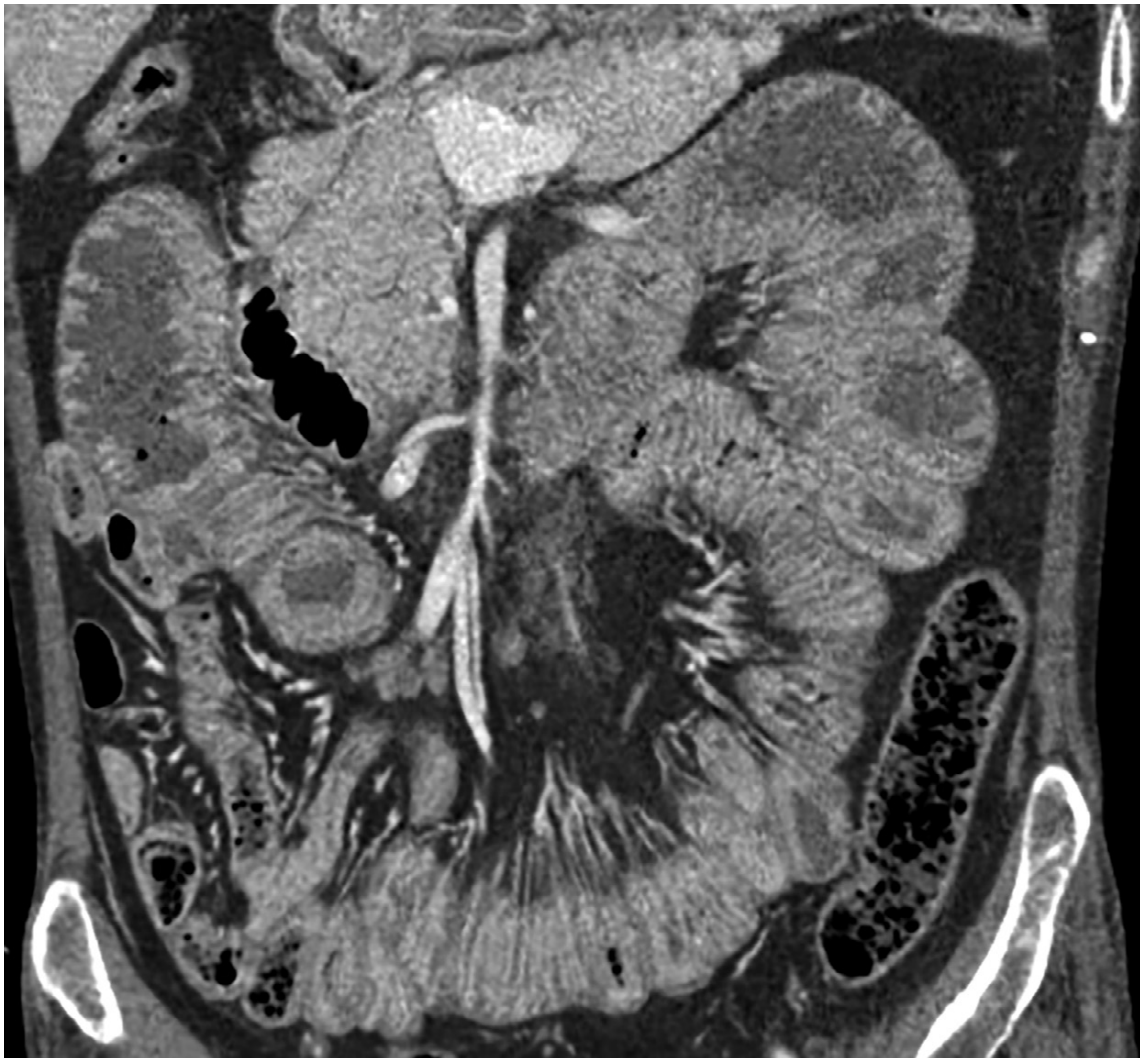
Рисунок 4. КТ брюшной полости: парез кишечника.

**Figure:**
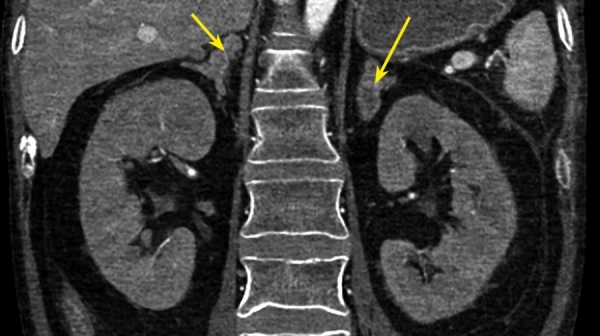
Рисунок 5. КТ брюшной полости: диффузно-гиперплазированные надпочечники: правый — до 1,4 см, левый до — 1,6 см.

Учитывая гиперплазию обоих надпочечников и рецидивирующую тяжелую гипокалиемию, выполнено обследование гипофизарно-адреналовой оси. Выявлено повышение уровня кортизола до 1613 нмоль/л (185–624) и АКТГ до 24,71 пмоль/л (1,03–10,74). Концентрация альдостерона и ренина, а также их соотношение — в норме. Учитывая интенсивную терапию, потребовавшую внутривенного введения дексаметазона до 32 мг в сутки, повышенный уровень АКТГ на этом фоне, проведение проб с подавлением АКТГ с использованием дексаметазона признано нецелесообразным.

При МРТ хиазмально-селлярной области с контрастированием данных за объемное образование гипофиза не получено.

Диагностирован эндогенный гиперкортицизм, АКТГ-эктопированный синдром. Наиболее вероятным источником избыточной продукции АКТГ признавалось визуализированное при КТ новообразование легкого. Другой возможной причиной гиперкортицизма, предположительно, являлась МКЩЖ.

Тяжелый гиперкортицизм явился причиной иммунодефицита, в первую очередь по клеточному типу, соответствовавший по клинической картине ВИЧ-инфекции 4В, не подтвержденной лабораторно. Выявлены положительные тесты на вирус Эпштейна-Барр, цитомегаловирус. Назначена противовирусная терапия (ганцикловир внутривенно капельно).

При контрольной КТ грудной клетки через 20 дней выявлено резкое увеличение размеров новообразования в S3 левого легкого с 2,5 см до 4,4 см, с активным накоплением контрастного вещества. В S3 правого легкого появилось новое образование 2,3х1,2 см со схожими характеристиками (рис. 6).

**Figure:**
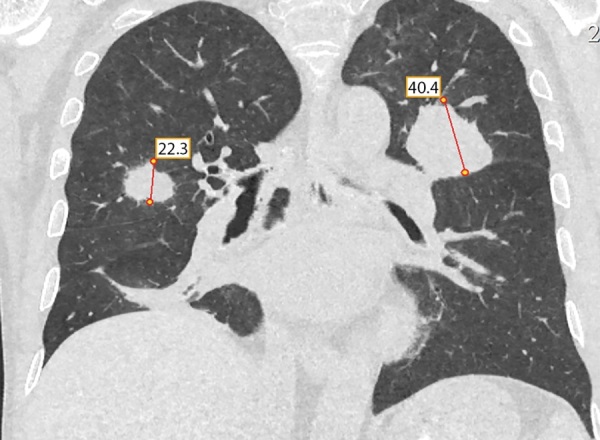
Рисунок 6. Контроль КТ грудной клетки через 20 дней: увеличение размеров новообразования в S3 левого легкого с 2,5 см до 4,4 см, накапливающее контраст, появление нового образования в S3 правого легкого 2,3х1,2 см.

Через 3 недели от начала интенсивной терапии (инфузионное введение препаратов калия, пероральный прием спиронолактона до 200 мг в сутки), на фоне стабилизации соматического статуса и после достижения стойкой нормокалиемии проведено оперативное лечение: тиреоидэктомия, центральная и правосторонняя боковая шейная лимфаденэктомия. Послеоперационный период протекал гладко. Голосовые связки после операции при ларингоскопии симметричны, подвижны в полном объеме. На первые сутки после операции уровень паратгормона — 2,4 пмоль/л (1–9), ионизированный кальций — 1,15 ммоль/л (1,12–1,32). Кальцитонин снизился до 126 пг/мл (<11,8). Состояние пациента улучшилось. Калий на фоне отмены препаратов калия и спиронолактона — 3,9 ммоль/л (3,5–5,1). Отмечено резкое снижение уровней кортизола и АКТГ в крови, что подтвердило предположение об эктопической гиперпродукции АКТГ клетками МКЩЖ. На первые сутки после операции уровень кортизола составил 145,85 нмоль/л (185–624), АКТГ — 3,03 пмоль/л (1,03–10,74). Кортизол на 6-е сутки после операции на фоне экзогенного введения 60 мг гидрокортизона — 533,93 нмоль/л (185–624), АКТГ — 3,9 пмоль/л (1,03–10,74), кальцитонин — 135 пг/мл (<11,8). Для профилактики развития надпочечниковой недостаточности в послеоперационном периоде проводилась терапия гидрокортизоном 80 мг в сутки (доза подобрана по клиническим и лабораторным показателям) с постепенным снижением до поддерживающей дозы 30 мг перорально в сутки.

При гистологическом исследовании: МКЩЖ — 5,0 см, солидного строения правой доли ЩЖ с распространенной инвазией капсулы последней, врастанием в окружающую жировую клетчатку, очаговой сосудистой инвазией. Кроме того, выявлена папиллярная микрокарцинома 0,5 см, tall cell вариант, перешейка ЩЖ. При иммуногистохимическом исследовании (ИГХ) опухолевые клетки интенсивно экспрессируют кальцитонин, часть клеток слабо и умеренно экспрессируют TTF 1, малая часть клеток экспрессируют цитокератин 19, тиреоглобулин, слабо фокусно экспрессируют АКТГ. Индекс пролиферативной активности Ki 67 — 5%. В центральной и боковой клетчатке шеи в 7 из 19 удаленных лимфоузлов выявлены метастазы МКЩЖ до 2,5 см с субтотальным вытеснением лимфоидной ткани и врастанием в окружающую жировую клетчатку.

Через 5 дней после операции при контрольной КТ грудной клетки в обоих легких визуализированы множественные шаровидные образования с признаками распада по типу абсцедирования, размером до 4,2 см (рис. 7). Рассеянные очаговые образования в обоих легких до 1 см; в S9,10 левого легкого пневмоническая инфильтрация легочной ткани по типу консолидации; в верхних долях диффузная очаговая интерстициальная инфильтрация.

**Figure:**
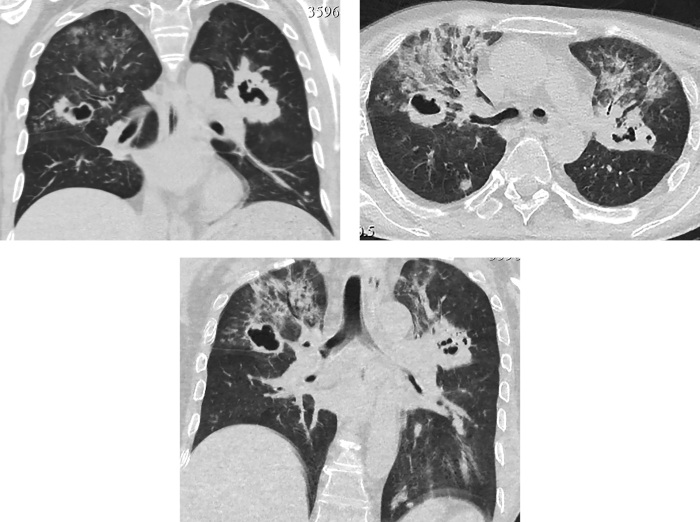
Рисунок 7. Контроль КТ грудной клетки через 5 дней после операции: в обоих легких множественные шаровидные образования с абсцедированием до 4,2 см. Рассеянные очаговые образования в обоих легких до 10 мм. В S9,10 левого легкого пневмоническая инфильтрация.

У пациента были взяты посевы, выполнена микроскопия и ПЦР на туберкулез. Все показатели оказались отрицательными. Туберкулез был исключен. По данным микроскопии с окрашиванием калькофлуором белым, теста на галактоманнан, посева на грибы диагностирован инвазивный аспергиллез с поражением обоих легких. Инициирована антимикотическая (вориконазол) и антибактериальная (ванкомицин, левофлоксацин) терапия, продолжена противовирусная терапия, гидрокортизон, отхаркивающая терапия, гепатопротекторная и противоязвенная терапия. На фоне проводимого лечения отмечалась значительная положительная динамика, как клинически, так и при КТ-контроле — через 9 дней отмечен регресс воспалительных изменений в обоих легких (рис. 8).

**Figure:**
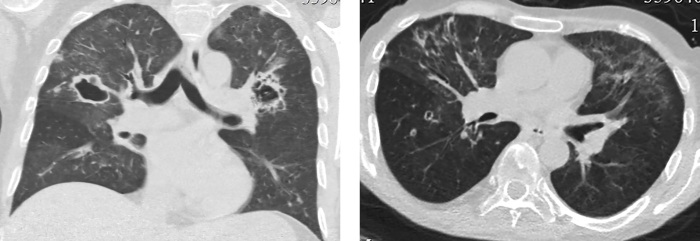
Рисунок 8. КТ грудной клетки через 9 дней после начала антимикотического лечения: регресс воспалительных изменений в легких.

Окончательный основной диагноз был сформулирован следующим образом: «Первично-множественная карцинома щитовидной железы: медуллярная карцинома щитовидной железы pT3aN1b(7/19)M0, стадия IVA. Папиллярная микрокарцинома щитовидной железы, tall cell вариант, pT1aN0M0, стадия I. Эндогенный гиперкортицизм. АКТГ-эктопированный синдром с эктопической продукцией АКТГ медуллярной карциномой щитовидной железы. Вторичная хроническая надпочечниковая недостаточность после устранения источника избыточной эктопической продукции АКТГ (тиреоидэктомия, центральная и боковая (справа) шейная лимфаденэктомия от 10.12.2020)».

Сопутствующие заболевания: инвазивный аспергиллез с поражением легких на фоне перенесенной билатеральной пневмонии, коронавирусной инфекции. Вторичный выраженный иммунодефицит. Сахарный диабет типа 2 на инсулинотерапии, компенсированный.

## Исход и результаты последующего наблюдения

От дальнейшего лечения в клинике пациент отказался по семейным обстоятельствам (необходимость приезда домой в канун Нового года), получил рекомендации о продолжении лечения в стационарных условиях и благополучно вернулся в родное село. Со слов родственников, в настоящее время явления тетрапареза у пациента регрессировали, ходит самостоятельно, все движения в полном объеме, полностью себя обслуживает, навещает родственников. Это свидетельствует о функциональном характере тетрапареза вследствие тяжелой гипокалиемии. К сожалению, нет никакой информации о контрольных результатах лабораторно-инструментальных методов обследований (недоступны ввиду отдаленности места жительства пациента).

## ОБСУЖДЕНИЕ

Примерно у 0,6% пациентов с МКЩЖ имеется АКТГ-эктопированный синдром, и на долю МК ЩЖ приходится примерно 2–7,5% случаев АКТГ-эктопированного синдрома [6]. Согласно описанным в литературе клиническим случаям МКЩЖ с эктопической гиперпродукцией АКТГ, средний возраст пациентов составлял 44 года; преобладают мужчины, соотношение мужчин и женщин — 2:1 [7][8]. У большей части пациентов встречаются спорадические случаи МКЩЖ, описаны единичные случаи наследственных форм в рамках синдромов МЭН 2A и МЭН 2В [9]. Прогноз больных с МКЩЖ существенно ухудшается при сочетании с гиперпродукцией АКТГ [10]. Примерно у половины описанных пациентов с МК ЩЖ с эктопической гиперпродукцией АКТГ на момент постановки диагноза имеются отдаленные метастазы, у 19% — метастазы в лимфатические узлы шеи. Наиболее частыми органами отдаленного метастазирования являются печень, а также легкие и кости. Почти для всех описанных больных был характерен тяжелый гиперкортицизм, с неподавляемым уровнем кортизола на фоне больших доз дексаметазона, с тяжелой и трудно корригируемой гипокалиемией.

Чаще всего симптомы МКЩЖ предшествуют развитию симптоматики эндогенного гиперкортицизма на несколько месяцев. Описаны случаи возникновения эндогенного гиперкортицизма через 20 лет после удаления первичной МКЩЖ, что говорит о возможности приобретения опухолевыми клетками способности вырабатывать АКТГ или КРГ [6]. У некоторых пациентов гиперкортицизм возникал одновременно с развитием метастазов. При МКЩЖ с АКТГ-эктопированным синдромом у 50% пациентов имеются отдаленные метастазы на момент постановки диагноза, тогда как для МКЩЖ без паранеопластического синдрома отдаленные метастазы характерны только в 10–15% случаев.

У большинства пациентов с эктопической секрецией АКТГ МКЩЖ при ИГХ выявляются АКТГ- или КРГ-позитивные клетки в удаленной опухоли. У некоторых все же подобного иммуногистохимического окрашивания не отмечается. Предполагается несколько возможных причин этому: 1) несмотря на повышенную секрецию, концентрация хранящихся запасов АКТГ или КРГ в опухолевой ткани низкая; 2) во внегипофизарных опухолях нарушены трансляция и процессинг мРНК проопиомеланокортина, в результате чего синтезируются предшественники АКТГ [6]. В силу этого гибридизация in situ мРНК проопиомеланокортина предложена в качестве полезного способа выявления источника эктопической продукции АКТГ.

Основными задачами лечения больных с АКТГ-эктопированным синдромом вследствие МКЩЖ являются: лечение первичной опухоли и одновременный контроль эндогенного гиперкортицизма, так как обусловленные им осложнения, включая тяжелую гипокалиемию, существенно ухудшают качество жизни и прогноз пациентов, а также нередко могут служить одной из основных причин летального исхода [11]. При невозможности удаления первичной опухоли вследствие тяжести состояния больного или с целью подготовки к оперативному лечению используют терапию ингибиторами стероидогенеза (метирапон, митотан, кетоконазол, этомидат) [8], в случае распространенного процесса с метастатической МКЩЖ прибегают к двусторонней адреналэктомии. Относительно недавно в лечении стали применять тирозинкиназные ингибиторы (вандетаниб [6], сорафениб, кабозантиниб [2], субитиниб, селперкатиниб [2]).

## ЗАКЛЮЧЕНИЕ

АКТГ-эктопированный синдром с эктопической продукцией АКТГ медуллярной карциномой ЩЖ является редким грозным синдромом. Тяжесть течения обуславливает необходимость ранней диагностики с целью улучшения прогноза. К сожалению, в большинстве случаев первичный диагноз устанавливается на этапе, когда уже имеются регионарные и/или отдаленные метастазы МКЩЖ, а также тяжелые проявления гиперкортицзма, в том числе гипокалиемия, общая и проксимальная мышечная слабость вплоть до развития тетрапареза, стероидный сахарный диабет. Накопление и обобщение данных о столь редких случаях позволяет повышать настороженность врачей различных специальностей на выявление возможной эктопической гиперпродукции нейроэндокринных опухолей.

## ДОПОЛНИТЕЛЬНАЯ ИНФОРМАЦИЯ

Источники финансирования. Работа выполнена по инициативе авторов без привлечения финансирования.

Конфликт интересов. Конфликт интересов отсутствует.

Участие авторов. Все авторы одобрили финальную версию статьи перед публикацией, выразили согласие нести ответственность за все аспекты работы, подразумевающую надлежащее изучение и решение вопросов, связанных с точностью или добросовестностью любой части работы.

Согласие пациента. Пациент добровольно подписал информированное согласие на публикацию персональной медицинской информации в обезличенной форме.
